# Expression of calcium release-activated and voltage-gated calcium channels genes in peripheral blood mononuclear cells is altered in pregnancy and in type 1 diabetes

**DOI:** 10.1371/journal.pone.0208981

**Published:** 2018-12-13

**Authors:** Amol K. Bhandage, Zhe Jin, Sergiy V. Korol, Atieh S. Tafreshiha, Priya Gohel, Charlotte Hellgren, Daniel Espes, Per-Ola Carlsson, Inger Sundström-Poromaa, Bryndis Birnir

**Affiliations:** 1 Department of Neuroscience, Uppsala University, Uppsala, Sweden; 2 Department of Molecular Biosciences, The Wenner-Gren Institute, Stockholm University, Stockholm, Sweden; 3 Department of Women’s and Children’s Health, Uppsala University, Uppsala, Sweden; 4 Department of Medical Cell Biology, Uppsala University, Uppsala, Sweden; Arizona State University, UNITED STATES

## Abstract

Calcium (Ca^2+^) is an important ion in physiology and is found both outside and inside cells. The intracellular concentration of Ca^2+^ is tightly regulated as it is an intracellular signal molecule and can affect a variety of cellular processes. In immune cells Ca^2+^ has been shown to regulate e.g. gene transcription, cytokine secretion, proliferation and migration. Ca^2+^ can enter the cytoplasm either from intracellular stores or from outside the cells when Ca^2+^ permeable ion channels in the plasma membrane open. The Ca^2+^ release-activated (CRAC) channel is the most prominent Ca^2+^ ion channel in the plasma membrane. It is formed by ORAI1-3 and the channel is opened by the endoplasmic reticulum Ca^2+^ sensor proteins stromal interaction molecules (STIM) 1 and 2. Another group of Ca^2+^ channels in the plasma membrane are the voltage-gated Ca^2+^ (Ca_V_) channels. We examined if a change in immunological tolerance is accompanied by altered ORAI, STIM and Ca_V_ gene expression in peripheral blood mononuclear cells (PBMCs) in pregnant women and in type 1 diabetic individuals. Our results show that in pregnancy and type 1 diabetes ORAI1-3 are up-regulated whereas STIM1 and 2 are down-regulated in pregnancy but only STIM2 in type 1 diabetes. Expression of L-, P/Q-, R- and T-type voltage-gated Ca^2+^ channels was detected in the PBMCs where the Ca_V_2.3 gene was up-regulated in pregnancy and type 1 diabetes whereas the Ca_V_ 2.1 and Ca_V_3.2 genes were up-regulated only in pregnancy and the Ca_V_1.3 gene in type 1 diabetes. The results are consistent with that expression of ORAI, STIM and Ca_V_ genes correlate with a shift in immunological status of the individual in health, as during pregnancy, and in the autoimmune disease type 1 diabetes. Whether the changes are in general protective or in type 1 diabetes include some pathogenic components remains to be clarified.

## Introduction

The immune system is inherently flexible. Both in pregnancy and with the onset of an autoimmune disease, an immunological shift takes place. In pregnancy, the immunological tolerance is expanded whereas in an autoimmune disease, it is decreased. How this plasticity comes about is still not clear despite intensive research [1–4]. The calcium (Ca^2+^) ion is an intracellular messenger in cells where it regulates multitude of mechanisms that in immune cells includes; activation, differentiation, proliferation, secretion and migration [5–11]. If calcium is involved in the change in immunological tolerance as observed during pregnancy and autoimmune diseases, then it is possible that expression of plasma membrane ion channels that regulate entry of the calcium ions into the cells may be altered when the immunological shift takes place resulting in altered expression in pregnancy and autoimmune diseases i.e. type 1 diabetes.

Ca^2+^ ions are present in both extra- and intracellular fluids in mammals. The intracellular concentration is tightly regulated. Increase of Ca^2+^ ion concentration in the cytoplasm of immune cells most often is associated with either release of Ca^2+^ from intracellular stores, like the endoplasmic reticulum (ER), or entry through ion channels in the plasma membrane. Store-operated Ca^2+^ entry (SOCE) through Ca^2+^ release-activated Ca^2+^ (CRAC) channels are present in many tissues including neurons, cardiac myocytes, skeletal muscle cells, pregnant human myometrium, vascular smooth muscle cells, pancreatic islet β cells, endothelial cells and most immune cells [6, 12–20] and are thought to be responsible for the majority of Ca^2+^ influx in at least the immune cells [6, 8]. Other Ca^2+^ permeable channels may be present in the immune cells like the voltage-gated Ca^2+^ channels (Ca_v_), glutamate-gated NMDA receptors, TRP channels and P2X receptors but generally less is known about the role of these channels in the immune system [8, 21–24]. The CRAC and some Ca_v_ channels are regulated by stromal interaction molecules (STIM1 and 2) which are located in the ER membrane [6, 8, 25–29].

The CRAC channels are tetramers of ORAI proteins that form the channel pore in the plasma membrane. The channels can be formed from homo- or heteromeric ORAI proteins (ORAI 1, 2 or 3) that differ in their kinetic properties [6, 9]. The store operated Ca^2+^ entry is initiated by STIM that are sensors for Ca^2+^ in the ER and when the ER Ca^2+^ concentration drops significantly, they cluster and at the ER-plasma membrane junction they bind to and open the CRAC channels. There are 10 members in the voltage-gated Ca^2+^ channel family [30]. In excitable cells the channels are opened by depolarization of the membrane potential but in immune cells additional mechanism involving STIM appears to participate in regulating the channels activation mechanism [25–27]. STIM 1 and STIM 2 are structurally similar molecules but STIM 2 has lower affinity for Ca^2+^ [31, 32].

We examined if the ORAI, Ca_v_ and STIM mRNAs expression in peripheral blood mononuclear cells (PBMCs) was correlated to altered immunity state in humans. We examined mRNAs isolated from PBMCs from healthy individuals, healthy pregnant women and individuals with type 1 diabetes. The results show that in pregnancy and in type 1 diabetes, the mRNA levels of genes encoding the CRAC channels and the Ca^2+^ sensing proteins were significantly altered. Ca_v_ channels were normally expressed at lower levels but significant changes were observed for specific L, R and T-type Ca^2+^ channels.

## Material and methods

### Study design

The studies were approved by the Regional Ethics Review Board in Uppsala. All individuals participating in the study were given oral and written information regarding the study and provided a written consent before entering the study. There were two groups in the study (A) healthy pregnant women and their age and body mass index (BMI) matched healthy control individuals including both men and non-pregnant women (S1 Table) and (B) type 1 diabetes individuals and their age, sex and BMI matched healthy control individuals (S1 Table). Both control groups were medical students, hospital employees and individuals recruited through poster advertising. Inclusion criteria for both control groups were self-reported physical health, no ongoing infection and no daily medication. None of the healthy controls had a first degree relative diagnosed with type 1 diabetes. Additional exclusion criteria for healthy control women were pregnancy, breast feeding and use of hormonal contraception. Pregnant participant in this study were recruited from women participating in a study at the Department for Women’s and Children’s Health, Uppsala University. They visited the lab between gestational weeks 36 and 41. Individuals with type 1 diabetes were recruited at the Department of Endocrinology and Diabetology and routine blood samples were analyzed at the department of Clinical Chemistry and Pharmacology, Uppsala University Hospital. Venous blood samples were collected into EDTA tubes for later isolation of PBMCs, see description below.

### PBMCs preparation

Blood samples were subjected to density gradient centrifugation to isolate PBMCs. In brief, samples were diluted in 1:1 ratio in MACS buffer (Miltenyi Biotec, Madrid, Spain) and layered on Ficoll-paque plus (Sigma-Aldrich, Hamburg, Germany). These diluted samples were centrifuged at 400g for 30 minutes at room temperature. The lymphocyte layer (PBMCs) was carefully withdrawn and washed twice in MACS buffer. PBMCs were saved in RNAlater (Sigma) at -80°C for later mRNA extraction.

### Total RNA isolation and real-time quantitative reverse transcription PCR

PBMC samples were processed for total RNA extraction using Gen Elute total RNA Miniprep (Sigma-Aldrich) or RNA/DNA/Protein Purification Plus Kit (Norgen Biotek, Ontario, Canada) and the concentration of total RNA was measured by Nanodrop (Nanodrop Technologies, Thermo Scientific, Inc., Wilmington, DE, USA). Further, 1.0–1.5 μg RNA was treated with 0.6 U DNAse I (Roche, Basel, Switzerland) for 30 minutes at 37°C, with 8 mM EDTA for 10 minutes at 75°C and then converted to cDNA using Superscript III or IV reverse transcriptase (Invitrogen, Stockholm, Sweden) in a 20 μl reaction. Reverse transcriptase negative control was performed in order to exclude genomic DNA contamination. Real-time quantitative PCR (RT-qPCR) was performed in 10 μl volume containing 4 μl cDNA (8–15 ng), 1×PCR reaction buffer, 3 mM MgCl_2_, 0.3 mM dNTP, 0.8 U JumpStart Taq DNA polymerase (Sigma-Aldrich), 1×ROX reference dye, 5×SYBR Green I (Invitrogen) and 0.4 μM each of forward and reverse primers. The gene-specific primer pairs (S2 Table) were designed using NCBI Primer-Blast, synthesized by Sigma Aldrich and further validated on human prefrontal cortex cDNA by the identification of the single peak in the melt curve and the single band of amplicon size on agarose gel. Amplification was performed in 384-well optical plates (Corning, 3757, Sigma-Aldrich) using the ABI PRISM 7900HT Sequence Detection System (Applied Biosystems, Stockholm, Sweden) with an initial denaturation of 5 min at 95°C, followed by 45 cycles of 95°C for 15 s, 60°C for 30s, and 72°C for 30 s, further followed by melt curve to ensure single product amplification. Cycle threshold (Ct) values were analyzed using SDS 2.4 and RQ Manager 1.2 softwares provided with the instrument. Since the expression of reference gene may differ between different cell types, it is very important to use validated and stable reference genes for normalization of qPCR data. We used importin 8 (IPO8) and TATA-binding protein (TBP) for normalization [33, 34]. The expression of each target gene relative to a normalization factor (geometric mean of two reference genes—IPO8 and TBP) was calculated with Data Assist v2.0 using the 2^−ΔCt^ method as previously described [35].

### Western blot analysis

Protein extraction from PBMC samples was performed using RNA/DNA/Protein Purification Plus Kit (Norgen Biotek, Ontario, Canada). Proteins were measured using the RC DCTM protein assay kit (Bio-Rad, USA) in Multiskan MS plate reader (Labsystems, Vantaa, Finland) and the concentration was calculated by plotting standard curve. Protein samples (20–60 μg) subjected to SDS-PAGE using 8% polyacrylamide gels and transferred to Amersham Hybond PVDF membranes by either wet or semi-dry transfer systems. The membranes were blocked with 10% FBS in Tris buffered saline containing 0.1% Tween (TBS-T) for 1 h and incubated overnight at 4°C with primary antibodies against STIM2 (1:200, Cell Signaling Technology, Cat No. 4917), ORAI1 (1:500, Alomone labs, Cat No. ACC-060), ORAI2 (1:500, Alomone labs, Cat No. ACC-061), Ca_V_1.3 (1:500, Alomone labs, Cat No. ACC-005), Ca_V_2.3 (1:500, Alomone labs, Cat No. ACC-006) and GAPDH (1:3000; Merck Millipore, Cat No. ABS16). After intensive washing with TBS-T, the membranes were further incubated with horseradish peroxidase-conjugated secondary antibody (1:3000; Cell Signaling Technology, Cat No. 7074) for 2 h. Further, membranes were washed intensively and developed for detection of immunoreactive protein bands using ECL Prime Western Blotting System (GE Healthcare, RPN2232). Bands were visualized in ChemiDoc^MP^ imaging system (Bio-Rad).

### Statistical analysis

Statistical analysis and data mining were done by using Statistica 12 (StatSoft Scandinavia, Uppsala, Sweden) and GraphPad Prism 7 (La Jolla, CA, USA). The statistical tests were performed after omitting outliers identified by Tukey test. The whiskers and the outliers are plotted by the Tukey method which uses +/- 1.5 inter-quartile distance i.e. interquartile range (IQR), the difference between the 25th and 75th percentiles. All data points were included when calculating IQR. The differences between groups were assessed by nonparametric Kruskal–Wallis ANOVA on ranks with Dunn’s post hoc test or by one-way ANOVA with Bonferroni post hoc test depending on the normality of the data. Normality of the data was determined by Shapiro-Wilk normality distribution test (S3 Table). A general stepwise linear regression model was used to identify covariates (e.g., age, gender and BMI). Variables with a significant association with groups were included in the final statistical model as covariates. The significance level was set to p < 0.05. We further correlated expression level of all CRAC and VDCC channel subunits with demographic characteristics of type 1 diabetic donors such as age at onset of T1D and duration of T1D. The correlation was accessed using non-parametric Spearman rank correlation.

## Results

The demographic characteristics of individuals in this study were shown in S1 Table. As expected, the blood glucose, C-peptide and HbA1c levels in type 1 diabetic individuals were significantly different when compared with healthy controls (S1 Table). There was no significant difference in age, BMI (controls A vs. pregnant women, and nondiabetic controls B vs. type 1 diabetic) and gender (nondiabetic controls B vs. type 1 diabetic). The mRNAs expression of three ORAI (1–3), STIM1 and 2 and ten voltage-gated Ca^2+^ channel-forming α1 subunits (Ca_V_1.1–1.4, Ca_V_2.1–2.3, Ca_V_3.1–3.3) in PBMCs was quantified by RT-qPCR in samples from controls (A) and (B), pregnant women and type 1 diabetic individuals. The primers covered all transcripts known today for the particular gene (S2 Table) and ORAI 1 mRNA expression was also tested by an additional pair of primers to verify the biological results obtained.

### The expression of ORAIs and STIM mRNAs in PBMCs is altered in pregnancy

**Fig 1:**
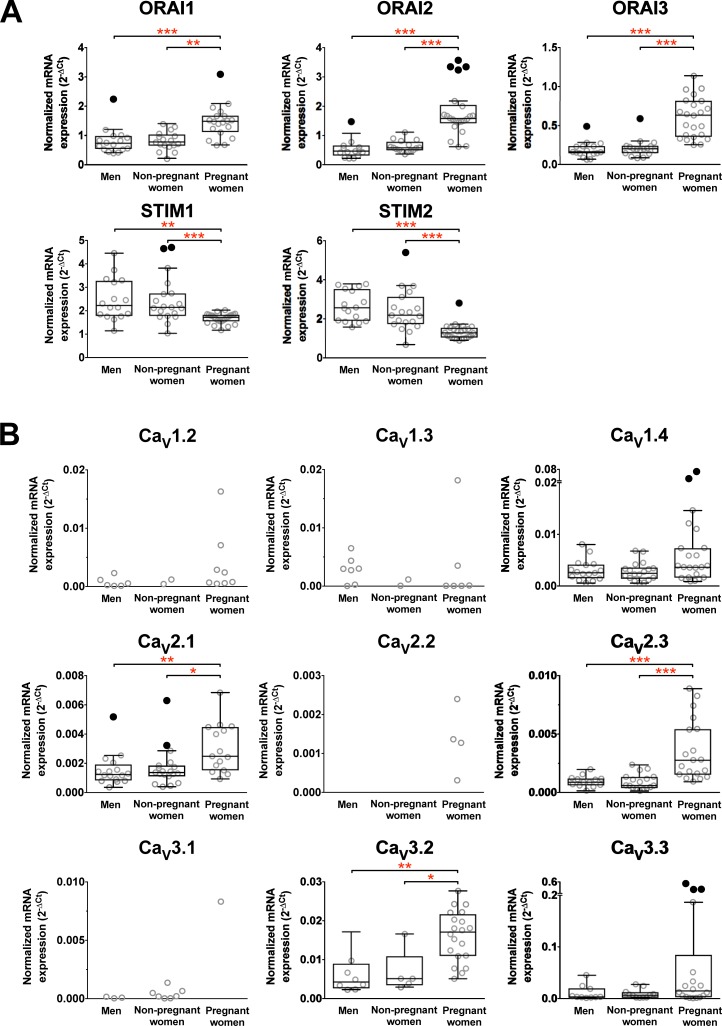
Altered mRNA expression of specific calcium release activated calcium (CRACs) channel and voltage-gated calcium channel (Ca_v_) subunits in PBMCs from non-pregnant controls and pregnant women. Data from each group is presented as scatter dot plot (°) or box and whiskers plot with median and whiskers plotted by Tukey method to determine outliers (• - above or below the whiskers). Ca_V_1.1 subunit mRNA was not detected in any sample. Statistical analysis was performed by excluding outliers depending on normality distribution of the data and only the subunits with statistically significant differences are mentioned below. One-Way ANOVA with Bonferroni post-hoc test: ORAI1, df = 48, p = 0.003; Kruskal–Wallis ANOVA on ranks with Dunn’s *post hoc* test: ORAI2, *H*_(1, 46)_ = 28.5, *p* < 0.001; ORAI3, *H*_(1, 54)_ = 37.2, *p* < 0.001; STIM1, *H*_(1, 58)_ = 18.3, *p* < 0.001; STIM2, *H*_(1, 58)_ = 32.5, *p* < 0.001; Ca_V_2.1, *H*_(1, 48)_ = 13.1, *p* < 0.001; Ca_V_2.3, *H*_(1, 49)_ = 22.1, *p* < 0.001; Ca_V_3.2, *H*_(1, 31)_ = 17.7, *p* < 0.001. ** *p* < 0.01, *** *p* < 0.001.

Comparison of PBMCs gene expression from controls and pregnant women is shown in Fig 1A and the number of individuals expressing the target mRNAs in Table 1. ORAI1, ORAI2, ORAI3, STIM1 and STIM2 were expressed in the majority of the PBMCs samples from both controls and pregnant women. Interestingly, the average expression level of ORAI1, ORAI2 and ORAI3 were significantly up-regulated in pregnant women whereas, in contrast, both STIM1 and STIM2 were down-regulated.

**Table 1:** Number of individuals expressing CRAC or Ca^2+^V channel mRNAs in PBMCs.

	Controls (A) (n = 35)	Pregnant Women (n = 24)	Controls (B) Nondiabetic individuals (n = 21)	Type 1 diabetic individuals (n = 33)
**CRAC**				
ORAI1	33	19	21	33
ORAI2	31	22	21	33
ORAI3	33	23	21	33
STIM1	35	24	21	33
STIM2	35	24	20	32
**VGCCs**				
Ca_V_1.1	0	0	0	8
Ca_V_1.2	8	8	9	17
Ca_V_1.3	9	6	13	18
Ca_V_1.4	33	23	20	28
Ca_V_2.1	25	15	11	18
Ca_V_2.2	0	4	5	2
Ca_V_2.3	30	19	17	25
Ca_V_3.1	10	1	2	1
Ca_V_3.2	13	20	18	23
Ca_V_3.3	21	18	13	21

### The expression of Ca_V_ 2 and 3 channel subtypes may be altered in pregnancy

PBMCs from control and pregnant women expressed 8 and 9 Ca_v_ subtypes, respectively, of the 10 known genes encoding the α1 pore-forming Ca_V_ (Fig 1B). The number of individuals expressing each subtype is given in Table 1. Ca_V_1.4, Ca_V_2.1, Ca_V_2.3 and Ca_V_3.3 were expressed in more than 50% of samples and Ca_V_3.2 was more prominently expressed by pregnant women than controls. The frequency of expression for the remaining subtypes was lower. The Ca_V_1.1 was not detected in any of the samples. The average mRNA expression level of Ca_V_2.1, Ca_V_2.3 and Ca_V_3.2 was significantly increased in pregnant women. The expression profile of the Ca_V_ channel-forming α1 subunit in PBMCs is consistent with that specific subtypes of voltage-gated Ca^2+^ channels respond to altered immunological status of the pregnant woman.

### The expression of ORAIs and STIM2 mRNAs in PBMCs is altered in type 1 diabetes

**Fig 2:**
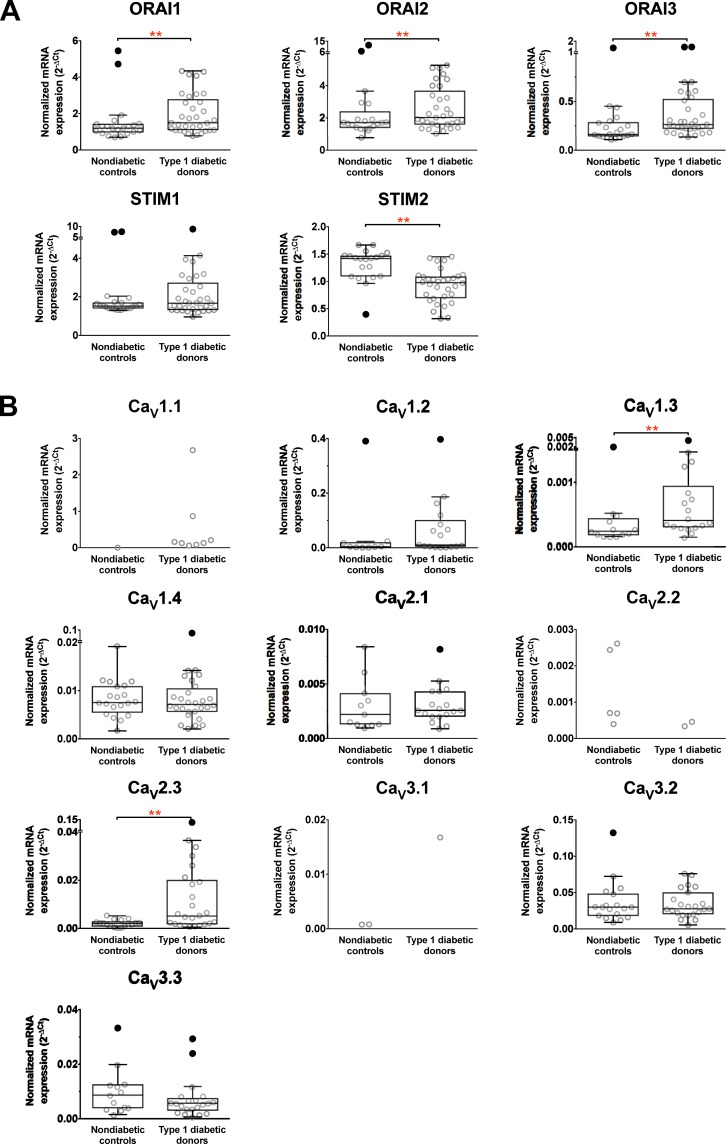
Altered mRNA expression of specific calcium release activated calcium (CRACs) channel and voltage-gated calcium channel (Ca_v_) subunits in PBMCs from non-diabetic controls and T1D patients. Data from each group is presented as scatter dot plot (°) or box and whiskers plot with median and whiskers plotted by Tukey method to determine outliers (• - above or below the whiskers). Statistical analysis was performed by excluding outliers depending on normality distribution of the data and only the subunits with statistically significant differences are mentioned below. One-Way ANOVA with Bonferroni post-hoc test: STIM2, df = 45, p = 0.002; Kruskal–Wallis ANOVA on ranks with Dunn’s *post hoc* test: ORAI1, *H*_(1, 47)_ = 9.1, *p* < 0.003; ORAI2, *H*_(1, 47)_ = 7.9, *p* < 0.005; ORAI3, *H*_(1, 48)_ = 8.2, *p* < 0.004; Ca_V_1.3, *H*_(1, 29)_ = 6.9, *p* < 0.008; Ca_V_2.3, *H*_(1, 41)_ = 7.4, *p* < 0.006. ** *p* < 0.01.

Comparison of PBMCs genes from nondiabetic and type 1 diabetic individuals is shown in Fig 2A and the number of individuals expressing the specific genes in Table 1. ORAI1, ORAI2, ORAI3, STIM1 and STIM2 were expressed in the majority of the samples from both nondiabetic and type 1 diabetic individuals. Similar to the expression in PBMCs from pregnant women, the average expression level of ORAI1, ORAI2 and ORAI3 were significantly up-regulated by type 1 diabetes. In contrast, the STIM2 mRNAs was down-regulated but the level of STIM1 gene expression was unaltered when samples from nondiabetic and type 1 diabetic individuals were compared.

### The expression of Ca_v_ 1 and 2 channel subtypes may be altered by type 1 diabetes

PBMCs from nondiabetic and type 1 diabetic individuals expressed 9 and 10 Ca_V_ subtypes, respectively, of the 10 known α1 pore-forming Ca_V_ genes (Fig 2B). The number of individuals expressing each subtype is given in Table 1. Ca_V_1.3, Ca_V_1.3, Ca_V_2.1, Ca_V_2.3, Ca_V_3.2 and Ca_V_3.3 were expressed in more than 50% of samples, Ca_V_1.2 was more prominently expressed in individuals with type 1 diabetes but for the remaining subtypes frequency of expression was lower. The average gene expression level of Ca_V_1.3 and Ca_V_2.3 was significantly increased in type 1 diabetes. The expression profile of the Ca_V_ channel-forming α1 subunit in PBMCs is consistent with that specific subtypes of voltage-gated Ca^2+^ channels respond to altered immunological status of individuals with type 1 diabetes.

### Type 1 diabetes duration and age of onset influences expression of specific channels

**Fig 3:**
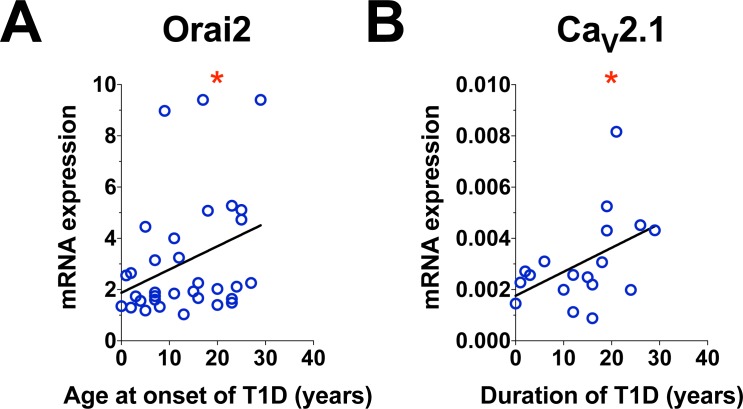
Correlation between the mRNA expression in PBMCs and demographic characteristics of type 1 diabetic donors. A. Correlation between ORAI2 and age at onset of type 1 diabetes. B. Correlation between Ca_V_2.1 and duration of Type 1 diabetes. The correlation was accessed using non-parametric Spearman rank test. * indicates p value < 0.05.

We examined if disease duration or age at onset affected the expression of the ORAIs, STIM1 and 2 and the Ca_v_1.2 channels. Significant correlation was found for ORAI2 with age of onset of type 1 diabetes (Fig 3A, *p* <0.05) and Ca_V_1.2 (Fig 3B, *p* <0.05) with duration of type 1 diabetes.

### STIM2, Cav1.3, Cav2.3 and ORAI1 and 2 proteins are expressed in PBMCs from controls and type 1 diabetic individuals

**Fig 4:**
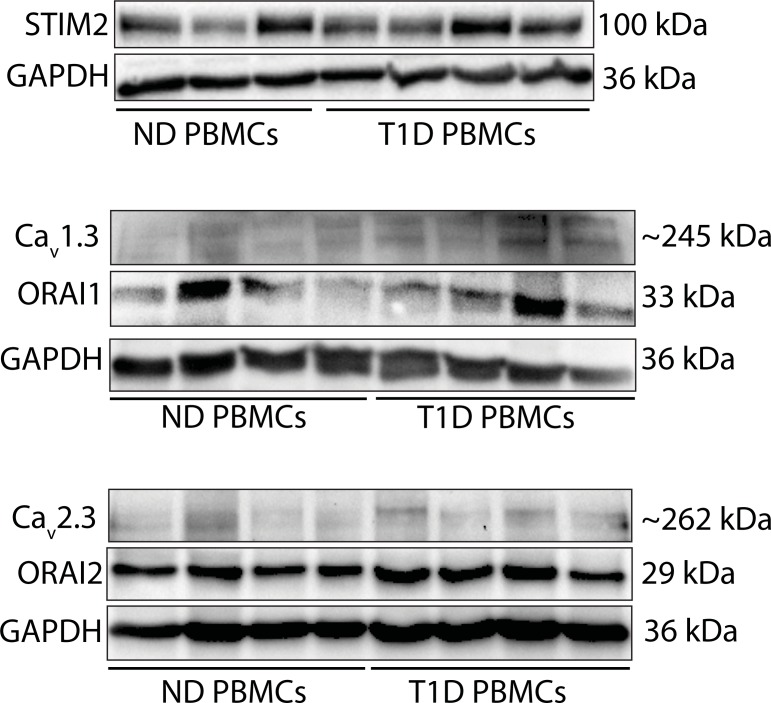
Western blot analysis. Western blot analysis showing the expression of Ca^2+^ sensor protein stromal interaction molecules (STIM) 2, voltage-gated Ca^2+^ (Ca_V_) channel subunits Cav1.3 and Cav2.3, and Ca^2+^ release-activated Ca^2+^ (CRAC) channel proteins ORAI1 and 2 in PBMCs from ND (n = 3 or 4) and T1D (n = 4) individuals. GAPDH (Glyceraldehyde 3-phosphate dehydrogenase) served as loading controls.

Western blot analysis showed that STIM2, Cav1.3, Cav2.3 and ORAI1 and 2 proteins were detected in PBMCs samples from nondiabetic (ND) individuals (controls B) and type 1 diabetic (T1D) individuals (Fig 4). Due to the limited protein sample size, we did not quantify the protein expression levels in these samples.

## Discussion

Here we examined if we could detect any differences in expression of the plasma membrane Ca^2+^ channels in immune cells in pregnancy and in an autoimmune disease, type 1 diabetes. In both cases an immunological shift takes place but the outcome in terms of health differs. In one case, pregnancy, the effects are beneficial whereas in the other, type 1 diabetes, the effects are detrimental and contribute to establishing the disease. Ca^2+^ is an important intracellular messenger and a wide variety of cellular functions depends on the dynamics of the intracellular Ca^2+^ concentration. Recent evidence has suggested associations of altered SOCE with altered diabetes complications in diabetic nephropathy, diabetic vasculopathy and diabetic cardiomyopathy [14] whereas in pregnancy, SOCE expressed in the pregnant preterm and term human myometrium is not altered by the onset of labor [18].

The most prominent Ca^2+^ channels in the plasma membrane of immune cells are the CRAC channels that are opened by the ER Ca^2+^ sensor proteins STIM1 and 2 [6]. In this study we showed that both in pregnancy and in type 1 diabetes the mRNAs encoding proteins forming the pore of the CRAC channels, the ORAI1-3, were up-regulated whereas the low-affinity Ca^2+^ sensor gene, STIM2, was down-regulated. In pregnancy, even the high Ca^2+^-affinity STIM1 was down-regulated. Interestingly, differential expression has been observed for the ORAI 1–3 and STIM 1 during T cells activation where the mRNAs were up-regulated [36] and in Sjögren’s syndrome, an autoimmune disease, where the STIM1 and 2 proteins were reduced [37]. The opposite effects on expression of the pore-forming and the gating partner of the store operated Ca^2+^entry in our study is intriguing. Since the stoichiometry of ORAI:STIM protein ratio determines the Ca^2+^ current size and inactivation properties, the altered expression level of the genes is bound to affect the cellular responses [11, 38]. Indeed, Ca^2+^ entry in PBMCs from patients with Sjögren’s syndrome was reduced as compared with cells from healthy controls [37]. Furthermore, in neutrophils, a loss of STIM2 has been associated with lower levels of cytokine production whereas STIM1 was required and enough for classical short-term neutrophil responses [32]. Together the results suggest that the cellular Ca^2+^ dynamics and thus intracellular signaling are, at least in part, regulated by the expression levels of the plasma membrane CRAC channels and the STIM proteins.

The contribution of the Ca_V_ channels to the Ca^2+^ influx into the cells and its effects on immunity is less well defined than that of the CRAC channels [7, 8, 21, 22]. The expression profile of the Ca_v_ pore-forming α1 subunit in the PBMCs in this study was consistent with expression of L-, P/Q-, R- and T-type voltage-gated Ca^2+^ channels [30] in the cells and included channels with high-to-low threshold for voltage activation. The Ca_v_2.3 gene (R-type) was up-regulated in pregnancy and in type 1 diabetes whereas the Ca_V_ 2.1 (P/Q-type) and Ca_V_3.2 (T-type) genes were up-regulated only in pregnancy and Ca_V_1.3 (L-type) gene in type 1 diabetes. Voltage-gated Ca^2+^ channels are generally opened by a depolarizing voltage change in excitable cells like neurons and some endocrine cells but how these channels operate in immune cells has been somewhat of an enigma [6]. Importantly, however, selective opening of depolarizing channels is possible in immune cells expressing ligand-gated channels, e.g glutamate receptors or GABA_A_ receptors [23, 24, 39, 40] and, in addition, the STIM proteins have been shown to modulate both L- and T-type Ca_V_ channels [25–27]. A reciprocal relationship has been proposed between Ca_V_ and ORAI regulated by the potency of TCR signaling [26].

Temporal and spatial organization of Ca^2+^ entry can be created by organization of specific Ca^2+^ microdomains in the cells and, thereby, enabling selective activation of specific intracellular signaling cascades. These domains may be created by differential location of ion channels and intracellular proteins organized into distinct macromolecular complexes for mediation of biological effects channeled through specific signaling pathway [41–44]. How a change in the expression of CRAC, STIM or Ca_V_ genes then leads to altered intracellular Ca^2+^ signaling and immune function remains to be elucidated. Our results indicate that the expression levels of these genes are associated with the immune status in health, during pregnancy, and in the autoimmune disease type 1 diabetes. Further studies are required on specific immune cell populations i.e. CD4^+^, CD8^+^, Treg, dendritic cells or macrophages, were channel function, intracellular Ca^2+^ concentration dynamics and concomitant activation of signaling cascades is coupled to specific function.

In conclusion, these studies will contribute to the understanding of how cellular Ca^2+^ fluctuations are related to the immune shift observed in pregnancy and type 1 diabetes. Whether the changes are in general protective or in type 1 diabetes include some pathogenic components remains to be clarified.

## Supporting information

S1 Table Demographic characteristics of the study groups, data are presented as mean (SEM) or frequency (%). (DOCX)

S2 Table Primers for RT-qPCR. (DOCX)

S3 Table Analysis of data distribution by Shapiro–Wilk normality test. (DOCX)
