# Supplementary material for: Expression of calcium release-activated and voltage-gated calcium channels genes in peripheral blood mononuclear cells is altered in pregnancy and in type 1 diabetes
Source: PLoS One. 2018 Dec 13;13(12):e0208981. doi: 10.1371/journal.pone.0208981 (PMC6292698; doi:10.1371/journal.pone.0208981)
Supplement: S1 Table — (DOCX) [file pone.0208981.s001.docx]

**S1 Table.** **Demographic characteristics of the study groups, data are presented as mean (SEM) or frequency (%)**

|  | **Controls (A)** | **Pregnant women** | **p values** |
| --- | --- | --- | --- |
| Age (years) | 30.1 (0.8) | 30.4 (0.9) | 0.79# |
| Men | 16 | - | <0.001## |
| Women | 19 | 25 |  |
| BMI (kg/m^2^) | 24.2 (0.5) | 22.6 (0.6) ^a^ | 0.053# |
| Breast-feeding | 0 (0%) | 0 (0%) | - |

|  | **Nondiabetic controls (B)** | **Type 1 diabetic** | **p values** |
| --- | --- | --- | --- |
| Age (years) | 25.4 (1.0) | 26.9 (0.9) | 0.27# |
| Men | 10 | 21 | 0.27## |
| Women | 11 | 12 |  |
| BMI (kg/m^2^) | 23.4 (0.9) | 24.7 (0.5) | 0.19# |
| Glucose (mmol/l) | 5.3 (0.1) | 11.6 (0.8) | <0.001# |
| C-peptide (nmol/l) | 0.6 (0.03) | 0.2 (0.05) | <0.001# |
| HbA1c (mmol/mol) | 30.8 (0.5) | 59.7 (2.4) | <0.001# |

^a^ based on reported pre-pregnancy weight

BMI: Body mass index

# Unpaired t-test

## Fisher's exact test
