# Supplementary material for: Expression of calcium release-activated and voltage-gated calcium channels genes in peripheral blood mononuclear cells is altered in pregnancy and in type 1 diabetes
Source: PLoS One. 2018 Dec 13;13(12):e0208981. doi: 10.1371/journal.pone.0208981 (PMC6292698; doi:10.1371/journal.pone.0208981)
Supplement: S2 Table — (DOCX) [file pone.0208981.s002.docx]

**S2 Table. Primers for RT-qPCR**

| **Genes** | **Primer** | **Sequence** | **Exon-Exon Junction Primer** | **Location of primer** | **Amplicon Size (bp)** |
| --- | --- | --- | --- | --- | --- |
| **Endogenous control** |  |  |  |  |  |
| TBP | Forward | GAGCTGTGATGTGAAGTTTCC | NO | Exon 6 | 117 |
|  | Reverse | TCTGGGTTTGATCATTCTGTAG |  | Exon 7 |  |
| IPO8 | Forward | GCAAAGGAAGGGGAATTGAT | NO | Exon 20 | 91 |
|  | Reverse | CGAAGCTCACTAGTTTTGACCC |  | Exon 21 |  |
| **CRACs** |  |  |  |  |  |
| ORAI1_H1 | Forward | CGCCATGGTGGCAATGGT | YES | Exon 1-2 | 127 |
|  | Reverse | GCTGATCATGAGCGCAAACA |  | Exon 2 |  |
| ORAI1_H2 | Forward | CATGGTGGCAATGGTGGAGGTG | YES | Exon 1-2 | 77 |
|  | Reverse | AGGCACTGAAGGCGATGAGCA |  | Exon 2 |  |
| ORAI2 | Forward | ATGGTGGCCATGGTGGAGGT | YES | Exon 3-4 | 79 |
|  | Reverse | TGCAGGCGCTGAAGGCAAT |  | Exon 4 |  |
| ORAI3 | Forward | AAGCTCAAAGCTTCCAGCCGC | NO | Exon 1 | 98 |
|  | Reverse | GGTGGGTACTCGTGGTCACTCT |  | Exon 2 |  |
| STIM1 | Forward | AAGATGACAGACCGGAGTC | YES | Exon 4 | 93 |
|  | Reverse | GTGATTATGGCGAGTCAAGAG |  | Exon 5-6 |  |
| STIM2 | Forward | CCAGGATAGCAGTGCACGAA | YES | Exon 4-5 | 139 |
|  | Reverse | ATCCAGTTATGAGGTGGGCG |  | Exon 5-6 |  |
| **VDCCs** |  |  |  |  |  |
| Ca_V_1.1 | Forward | GATGACGAGGAAGATGAGCC | NO | Exon 17 | 116 |
|  | Reverse | AAGATGAAGAAGGAGCTGGC |  | Exon 17 |  |
| Ca_V_1.2 | Forward | TTCCAACCTGGAACGAGTGG | YES | Exon 3-4 | 101 |
|  | Reverse | AGGCATTGGGGTGAAAGAGG |  | Exon 4 |  |
| Ca_V_1.3 | Forward | GGTGATCCCCTTCCCCATTC | YES | Exon 1 | 162 |
|  | Reverse | ATAGTTTGCCTCGTTCGCGT |  | Exon 1-2 |  |
| Ca_V_1.4 | Forward | CCTGGGATCCGACATGGAAG | YES | Exon 6-7 | 89 |
|  | Reverse | ACTCAGTCTGGTTCAGCGTG |  | Exon 7 |  |
| Ca_V_2.1 | Forward | TTCAACATCGTCTTCACCTC | YES | Exon 30 | 81 |
|  | Reverse | GCGGAAATAATTCAGAATCCC |  | Exon 30-31 |  |
| Ca_V_2.2 | Forward | GGAATGTCTTTGACTTTGTCAC | YES | Exon 31 | 81 |
|  | Reverse | TGAAATTGTTCGTTTCCGC |  | Exon 31-32 |  |
| Ca_V_2.3 | Forward | GATGGGACTCCTTCGGCAAA | YES | Exon 15 | 71 |
|  | Reverse | CCCGTCAGGATCTGGAACAC |  | Exon 15-16 |  |
| Ca_V_3.1 | Forward | CCACGTGGTCCTTGTCATCA | YES | Exon 19 | 98 |
|  | Reverse | GGGTCAGGAAGATGCGTTCA |  | Exon 19-20 |  |
| Ca_V_3.2 | Forward | TCGAGGAGGACTTCCACAAG | NO | Exon 15 | 176 |
|  | Reverse | TGCATCCAGGAATGGTGAG |  | Exon 16 |  |
| Ca_V_3.3 | Forward | AGGATGAGCTATGACCAGCG | NO | Exon 16 | 151 |
|  | Reverse | CAGAGAGCAGGGACTCATGC |  | Exon 17 |  |
