# Supplementary material for: Expression of calcium release-activated and voltage-gated calcium channels genes in peripheral blood mononuclear cells is altered in pregnancy and in type 1 diabetes
Source: PLoS One. 2018 Dec 13;13(12):e0208981. doi: 10.1371/journal.pone.0208981 (PMC6292698; doi:10.1371/journal.pone.0208981)
Supplement: S3 Table — (DOCX) [file pone.0208981.s003.docx]

**S3 Table. Analysis of data distribution by Shapiro–Wilk normality test**

|  | **Pregnant women donors** | **Type 1 diabetic**  **donors** |
| --- | --- | --- |
| ORAI1 | 0.073 | < 0.001 |
| ORAI2 | < 0.001 | < 0.001 |
| ORAI3 | < 0.001 | < 0.001 |
| STIM1 | < 0.001 | < 0.001 |
| STIM2 | < 0.001 | 0.102 |
| Ca_V_1.1 | - | - |
| Ca_V_1.2 | < 0.001 | < 0.001 |
| Ca_V_1.3 | 0.008 | < 0.001 |
| Ca_V_1.4 | < 0.001 | 0.139 |
| Ca_V_2.1 | < 0.001 | 0.0042 |
| Ca_V_2.2 | - | - |
| Ca_V_2.3 | < 0.001 | < 0.001 |
| Ca_V_3.1 | **-** | - |
| Ca_V_3.2 | 0.021 | 0.005 |
| Ca_V_3.3 | < 0.001 | 0.008 |

P>0.05 indicates the data were normally distributed and thus, treated by one-way ANOVA followed by *Bonferroni* *post hoc* test and p<0.05 indicates the data were not normally distributed and thus, treated by Kruskal–Wallis ANOVA on ranks followed by Dunn’s *post hoc* test. Empty cells indicate that normality not determined due to few samples expressing that subunit and thus, no statistics performed.
